# Bismuth‐Tin Core–Shell Particles From Liquid Metals: A Novel, Highly Efficient Photothermal Material that Combines Broadband Light Absorption with Effective Light‐to‐Heat Conversion

**DOI:** 10.1002/advs.202407771

**Published:** 2024-10-07

**Authors:** Dogu Seyda, Orcun Dincer, Duygu İnce, Murathan Cugunlular, Husnu Emrah Unalan, Simge Çınar Aygün

**Affiliations:** ^1^ Department of Metallurgical and Materials Engineering Middle East Technical University (METU) Ankara 06800 Türkiye; ^2^ Present address: Department of Chemical and Materials Engineering Concordia University Montreal Quebec H3G 1M8 Canada

**Keywords:** bismuth‐tin (BiSn), broadband absorption, liquid metal particles, near‐infrared (NIR) light response, photothermal conversion, solar energy conversion

## Abstract

This study presents a pioneering investigation of hybrid bismuth‐tin (BiSn) liquid metal particles for photothermal applications. It is shown that the intrinsic core–shell structure of liquid metal particles can be instrumentalized to combine the broadband absorption characteristics of defect‐rich nano‐oxides and the high light‐to‐heat conversion efficiency of metallic particles. Even though bismuth or tin does not show any photothermal characteristics alone, optimization of the core–shell structure of BiSn particles leads to the discovery of novel, highly efficient photothermal materials. Particles with optimized structures can absorb 85% of broadband light and achieve over 90% photothermal conversion efficiency. It is demonstrated that these particles can be used as a solar absorber for solar water evaporation systems owing to their broadband absorption capability and become a non‐carbon alternative enabling scalable applications. We also showcased their use in polymer actuators in which a near‐infrared (NIR) response stems from their oxide shell, and fast heating/cooling rates achieved by the metal core enable rapid response and local movement. These findings underscore the potential of BiSn liquid metal‐derived core–shell particles for diverse applications, capitalizing on their outstanding photothermal properties as well as their facile and scalable synthesis conditions.

## Graphical abstract

This study introduces the liquid metal‐derived bismuth‐tin (BiSn) core–shell particles as novel photothermal materials. The unique structure combines the broadband absorption of a defect‐rich SnO shell with the high heat conversion of BiSn core. These particles outperform with their remarkable photothermal conversion efficiencies and fast responsiveness, thus opening the way for breakthroughs in solar energy systems and NIR‐sensitive technologies.

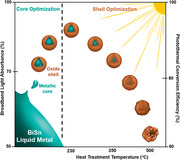

## 1 Introduction

The Earth is annually exposed to significant fluxes of solar energy, primarily in the near‐infrared (NIR) and visible spectrum.^[^ ^1^ ^]^ This readily available energy source has triggered the need to develop highly efficient photothermal materials capable of absorbing and converting solar energy into other useful forms of energy or to do work.^[^ ^1^, ^2^ ^]^ These materials hold significant promise across two primary domains: first, leveraging their broad‐spectrum light absorption for solar applications encompassing renewable solar energy, solar water evaporation, and steam generation.^[^ ^2^, ^3^, ^4^ ^]^ and second, benefiting from photo‐responsiveness, i.e., their ability to convert incident light into heat energy, for applications in diverse fields including photothermal therapy,^[^ ^5^, ^6^, ^7^ ^]^ NIR‐triggered soft actuators,^[^ ^8^, ^9^, ^10^, ^11^ ^]^ and photothermal catalysis.^[^ ^12^, ^13^ ^]^

Ideally, photothermal materials should have the ability to absorb light across the entire solar spectrum, spanning from 250 to 2500 nm, and efficiently convert a significant portion of this energy into heat.^[^ ^14^ ^]^ The first generation of photothermal materials includes plasmonic noble metal nanoparticles, carbon‐based nanoparticles, and semiconductor nanoparticles. Among them, metals and semiconductors show relatively high light‐to‐heat conversion efficiency; however, their light absorption capability is restricted to specific wavelengths; thus they can only harvest a portion of the available solar energy.^[^ ^6^, ^14^, ^15^, ^16^, ^17^, ^18^ ^]^ Carbon‐based nanoparticles, on the other hand, can exhibit broadband light absorption, but their photothermal conversion performance decays over time due to the aging instability of their molecular bonds.^[^ ^14^, ^19^ ^]^

One common approach to achieve enhanced light absorption is to collect the materials with different light absorption characteristics in one batch,^[^ ^4^, ^16^ ^]^ while the other innovative approach is to design novel composite materials hybridizing the complementary or synergistic optical properties.^[^ ^14^, ^20^, ^21^, ^22^, ^23^ ^]^ For instance, nano‐oxides were engineered by including defects or dopants in such a way to exhibit broadband absorption (e.g., black titania,^[^ ^17^, ^18^ ^]^ black tin oxide^[^ ^24^ ^]^), but again, their photothermal conversion efficiencies (PCEs) remained low. All in all, materials reported in the literature exhibit either broadband absorption or high light‐to‐heat conversion efficiency, but not both, with possibly only one exception of Ti_2_O_3_ nanoparticles, which exhibited light absorption capability between 250 and 2500 nm in addition to their PCE of ≈90%.^[^ ^25^, ^26^ ^]^

In addition to the light‐harvesting and light‐to‐heat conversion abilities, the heat transfer capability of the material is a third key parameter to obtain the nearly complete conversion of solar to thermal energy. However, this parameter seems to be overlooked in the design of photothermal materials. Examining the broadband absorber materials, it was interesting to realize that they are commonly made of materials with intrinsically low heat transfer coefficients like metal oxides and conventional carbon‐based materials.^[^ ^27^, ^28^, ^29^ ^]^ Thermal conductivity of such materials can be enhanced using defects.^[^ ^30^ ^]^ Even though the defect‐rich metal oxides exhibit higher thermal conductivity than their stoichiometric forms (e.g., 1.62–2.19 Wm^−1^ K^−1^ for Ti_2_O_3_^[^ ^30^ ^]^ as compared to 0.40–0.84 Wm^−1^ K^−1^ for TiO_2_^[^ ^31^ ^]^), these values still remain low compared to intrinsic thermal conductivities of metals (e.g., 19.00 Wm^−1^ K^−1^ BiSn at eutectic composition^[^ ^32^ ^]^). In addition, metallic materials’ heat capacities are commonly lower than their oxide counterparts (e.g., 683 J kg^−1^K^−1^ for TiO_2_,^[^ ^33^ ^]^ 786 J kg^−1^K^−1^ for Ti_2_O_3,_^[^ ^26^ ^]^ 710 J kg^−1^K^−1^ for carbon/graphite^[^ ^33^ ^]^ whereas 380 J kg^−1^K^−1^ for Au^[^ ^34^ ^]^ and 210 J kg^−1^K^−1^ for BiSn^[^ ^32^ ^]^ at eutectic composition), enabling them to achieve much higher temperatures with the same heat input. The higher the material temperature is, the faster cooling will be because of the increased driving force. Therefore, using materials with higher thermal conductivity and/or lower heat capacities would be expected to improve the solar‐thermal conversion efficiencies. In this study, we hypothesized that a core–shell structure, featuring a thermally conductive metallic core with low heat capacity and a broadband‐absorbing oxide shell, could integrate all these characteristics into a single hybrid material.

Liquid metal particles, an emerging class of functional materials, possess intrinsic core–shell structures due to their fabrication technique. Mechanical agitation of the liquid alloy forms metal/alloy particles encapsulated by a thin protective metal oxide layer.^[^ ^35^ ^]^ Unlike the core–shell materials reported in the literature, this facile one‐step method not only eases production but also eliminates fabrication‐related issues such as lattice mismatch between the core and the shell.^[^ ^35^, ^36^ ^]^ Having such a structure enables their fabrication from bulk liquid metal alloys, facilitates their dispersion in liquid media,^[^ ^37^ ^]^ and protects the metal core from corrosion over time.^[^ ^38^ ^]^ Among other unique properties, liquid metal particles have recently been investigated for their interactions with light. These studies commonly aimed to utilize gallium (Ga)‐based liquid metal particles for biomedical theranostic and reported PCEs of unmodified (as produced) liquid metal particles 15.5% for GaIn (at 1064 nm),^[^ ^39^ ^]^ 52% for GaInSn (at 785 nm).^[^ ^40^ ^]^ Moreover, comparable and enhanced efficiencies were reported when the liquid metal particles were modified with different photothermal nanomaterials, such as 39% for GaIn@Pt (at 1064 nm),^[^ ^39^ ^]^ 42.5% for Ga@rGO,^[^ ^41^ ^]^ and 65%,^[^ ^5^ ^]^ and 87% for Au nanoparticles on EGaIn surface (at 808 nm) in aqueous suspensions.^[^ ^42^ ^]^ Even though these PCEs are comparable or reasonably high to the traditional photothermal agent gold nanorods (21%),^[^ ^43^ ^]^ there is still room for enhancement in both the PCE and photothermal stability of liquid metal particles. Moreover, Ga‐based photothermal systems exhibit light absorption only in UV and visible light regions; therefore, their applicability for solar energy‐assisted photothermal systems is currently limited.^[^ ^7^ ^]^

Most liquid metal studies concentrate on Ga‐based particles because their melting points are sufficiently low to maintain an equilibrium liquid state at room temperature.^[^ ^44^ ^]^ However, recent studies have shown that the synthesis of undercooled liquid metal particles in high yield is also possible by eliminating highly potent nucleation sites in the constrained volume of particles.^[^ ^45^ ^]^ Hence, these particles can remain in a metastable undercooled state even at temperatures significantly below their melting point, allowing for the potential expansion of the liquid metal particle library.^[^ ^45^, ^46^ ^]^ Our previous study successfully demonstrated the straightforward fabrication of BiSn liquid metal particles.^[^ ^46^ ^]^ When Bi is alloyed with Sn during liquid metal particle synthesis, the resulting shell primarily consists of SnO due to the prevailing reaction environment.^[^ ^36^, ^46^, ^47^ ^]^ SnO has an inherently oxygen‐deficient structure,^[^ ^48^ ^]^ and this defect‐rich shell is expected to display optically “black” behavior.^[^ ^24^, ^49^ ^]^ This feature should facilitate broadband light absorption via enhanced light scattering and photothermal conversion via defect‐assisted energy transfer to lattice vibrations, i.e., heat in the BiSn liquid metal particles, even in their as‐produced state. Therefore, although Bi, Sn, or their oxides have not been reported to show any photothermal characteristics, it is hypothesized that BiSn liquid metal particle‐based materials show promise for photothermal applications benefiting from solar light. Moreover, control over nucleation and growth processes in particles through heat treatments was reported to allow manipulation of the core and shell phases.^[^ ^46^ ^]^ Therefore, resulting variations in their compartmentalization, thicknesses, and compositions offer a versatile tool for an in‐depth investigation of the relations between the structure and the photothermal characteristics of particles.

In this study, our objective was to comprehensively characterize the photothermal characteristics of BiSn liquid metal particle deposited layers (LMP). We found that these particles exhibited broadband light absorption, a feature which, to the best of our knowledge, has not been reported previously. Benefiting from the metastable nature of undercooled liquid metal particles, we conducted a comparative analysis of the light absorption properties between the liquid metal particles and their solidified counterparts, thereby elucidating the impact of core structure on their optical behavior. Then, we calculated the cooling rates and the PCEs of the BiSn core–shell particles. Furthermore, we heat‐treated the particles to tune their shell and investigated the influence of the shell layer on the photothermal properties of BiSn core–shell particles. Consequently, BiSn liquid metal particles and their derivatives have emerged as promising photothermal materials due to their broadband light absorption, high PCEs, and the tunability of these properties. In proof‐of‐concept studies, we highlighted two ideal characteristics of photothermal BiSn core–shell particles. First, we demonstrated their efficacy as solar absorbers, benefiting from their broadband light absorption. Second, we showed their utility as low thermal expansion and photothermal layers in an NIR‐triggered polymer actuator, benefiting from their photoactive response to NIR light.

## 2 Results and Discussion

### 2.1 Light Absorption Characteristics of the BiSn Core–Shell Particle Depositions

**Figure 1:**
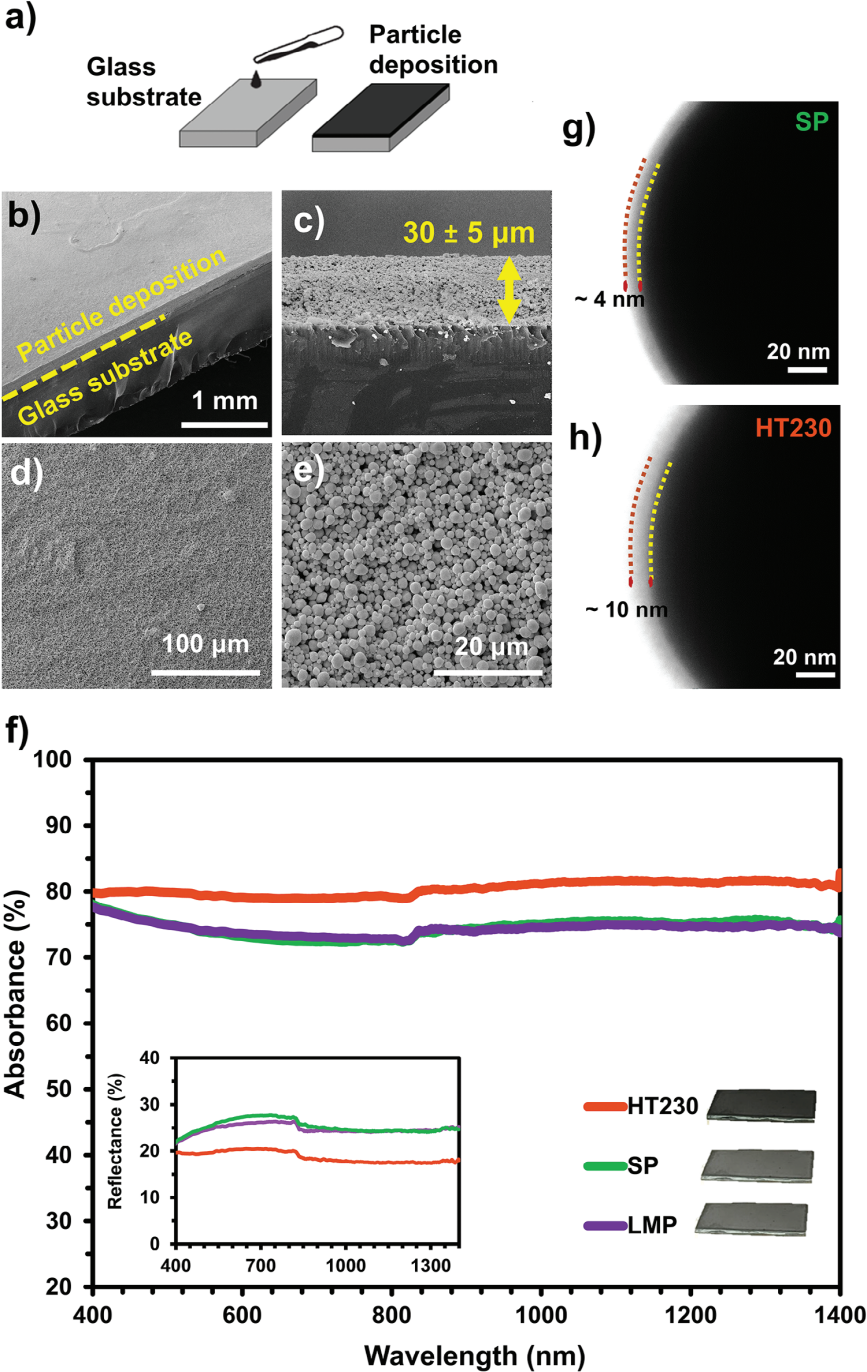
a) Schematic showing the preparation of particle depositions via drop‐casting on glass. SEM micrographs showing b) particle layer on glass (dashed line indicating the interface), c) coating thickness on the glass substrate (arrow indicating the thickness), d) particle deposition at low magnification, and e) particle deposition at high magnification, f) total absorbance and total reflectance (as an inset) spectra of HT230, SP, and LMP between 400 and 1400 nm. Digital photographs of the samples are given adjacent to the legend. HR‐TEM micrographs show the core–shell structure of g) solidified particles of SP and h) heat‐treated particles of HT230. Dashed lines in g and h indicate the boundaries of the shell layer.

The light absorption characteristics of the BiSn core–shell particles were evaluated using particle‐deposited glass substrates. The deposition was practiced by drop‐casting of BiSn particles dispersed in ethanol (**Figure** **1**a) and characterized by Scanning Electron Microscopy (SEM) analysis (Figure 1b–e). The micrograph in Figure 1b clearly shows the presence of highly packed and uniform particles on glass. The oxide shells around metallic particles enabled the homogeneous dispersion of BiSn particles in ethanol (Figure S1, Supporting Information) therefore promoting this highly packed and uniform deposition. The thickness of the particle layer was measured as 30 ± 5 µm using the cross‐sectional SEM micrograph provided in Figure 1c. The surface of the glass was entirely covered with particles, as confirmed by analyses at higher magnifications (Figure 1d,e). The coverage was extensive enough that the measured light transmittance through the coating was as low as 2% within the wavelength range of 200–1400 nm (Figure S2, Supporting Information).

Figure 1f shows the absorption spectrum of the heat‐treated BiSn core–shell particles at 230 °C (HT230), BiSn core–shell particles with solid state cores (SP), and BiSn liquid metal particles (LMP) within the wavelength range of 400–1400 nm. All the particles showed continuous (flat) absorption throughout the spectrum. This absorption is at the level of 80% for the HT230 and 75% for both the SP and LMP in the entire wavelength range of the measurements. The broadband absorption characteristics of the particles were revealed by their dark gray color, as observed in the photographs presented in the inset of Figure 1f. The higher absorbance of the HT230 was evident from its darker appearance. While the reflectance of the LMP and SP were close to 30% in the visible part of the spectrum, this value was only 20% for the HT230. The lower reflectance of HT230 further confirmed that the dark color stemmed from the high light absorption ability.

When comparing the optical properties of the particle depositions on glass, the similar behavior exhibited by both the LMP and SP suggested that the phase of the metal core, whether in a liquid or solid state, did not result in a significant difference. In contrast, the light absorbance of the HT230 was 5% higher than that of SP. Considering that the only difference between these samples is the oxide shell thickness and the increased amount of SnO, the oxide shell of particles appears to influence the light absorption characteristics of the particle deposited layer. High‐Resolution Transmission Electron Microscopy (HR‐TEM) analysis results comparing the solidified particles of SP and heat‐treated particles of HT230 are presented in Figure 1g,h, respectively, where dark regions indicate the core of the particles encapsulated with an oxide shell, observable as gray regions around the core. A more detailed analysis of these particles and their shell structure can be found in previous work.^[^ ^46^ ^]^ As SnO possesses a structure with a high density of defects,^[^ ^36^, ^50^, ^51^ ^]^ the enhanced broadband light absorption can be attributed to the augmented presence of a defect‐rich phase in these particles. It is well‐established that defects,^[^ ^17^, ^18^ ^]^ particularly oxygen vacancies, are responsible for decreased light scattering, which, in turn, can lead to an optical phenomenon known as “black” behavior characterized by broadband light absorption. Furthermore, the phenomena of internal light reflections between core and shell materials are known to result in continuous absorption of broadband spectrum and are used as electromagnetic wave absorber materials.^[^ ^52^ ^]^ Since BiSn core–shell particles are metal and metal oxide hybrids, light reflections between core and shell materials may also contribute to their light absorption capabilities reported in this work.

It's important to note that the SnO phase is inherently unstable under normal conditions.^[^ ^50^ ^]^ However, it attains stability when formed on the surface of the BiSn alloy at its eutectic composition.^[^ ^36^, ^47^ ^]^ Considering its nanoscale thickness when formed as a protective oxide layer, it can be deduced that a defect‐rich nano‐oxide shell with broadband light absorbance character can be obtained, especially when the particles are derived from BiSn liquid metal particles. Moreover, the size of the particles used here is in the submicron range (Figure S1b, Supporting Information), and it is known from the literature that it is possible to synthesize liquid metal nanoparticles^[^ ^53^ ^]^ and the light absorption ability is expected to be enhanced by reducing the particle size to the nanometer range.^[^ ^26^, ^39^, ^43^ ^]^

The solar energy is not evenly distributed throughout the spectrum. While the UV range (<400 nm) carries only 7% of the total solar energy, visible (400–760 nm) and infrared (>760 nm) ranges carry 50% and 43% of the solar energy, respectively.^[^ ^26^ ^]^ Broadband light absorbers have the potential to absorb the entire spectrum, thus getting the most out of the available solar energy source. Yet, the examples of such particles (e.g., black titania,^[^ ^18^, ^54^ ^]^ black tin oxide,^[^ ^24^ ^]^ Ti_2_O_3_^[^ ^25^, ^26^ ^]^) are only a few. Even though the broadband absorption characteristics were previously reported for black tin oxide, their absorbance decays through the NIR region.^[^ ^24^, ^55^ ^]^ Therefore, the continuous (flat) broadband light absorbance character of hybrid BiSn core–shell particles is novel.

### 2.2 Photothermal Conversion Characteristics of BiSn Core–Shell Particle Depositions

In addition to being an excellent broad‐spectrum light absorber, an ideal photothermal material should convert light into heat efficiently.^[^ ^14^ ^]^ Equation (1) shows the relationship between PCE (η) and material characteristics, where *hs* is the heat transfer coefficient of the illumination area, Δ*T* is the temperature difference between the maximum temperature of the material and the surrounding temperature, *I* is the irradiation power and *A*_λ_ is the normalized absorbance of the particle depositions at the irradiation wavelength. The detailed derivation of the equation and calculations are provided in the Supporting Information.

(1) $$\eta =\frac{hs\Delta T}{IA_{\lambda }}\;$$

**Figure 2:**
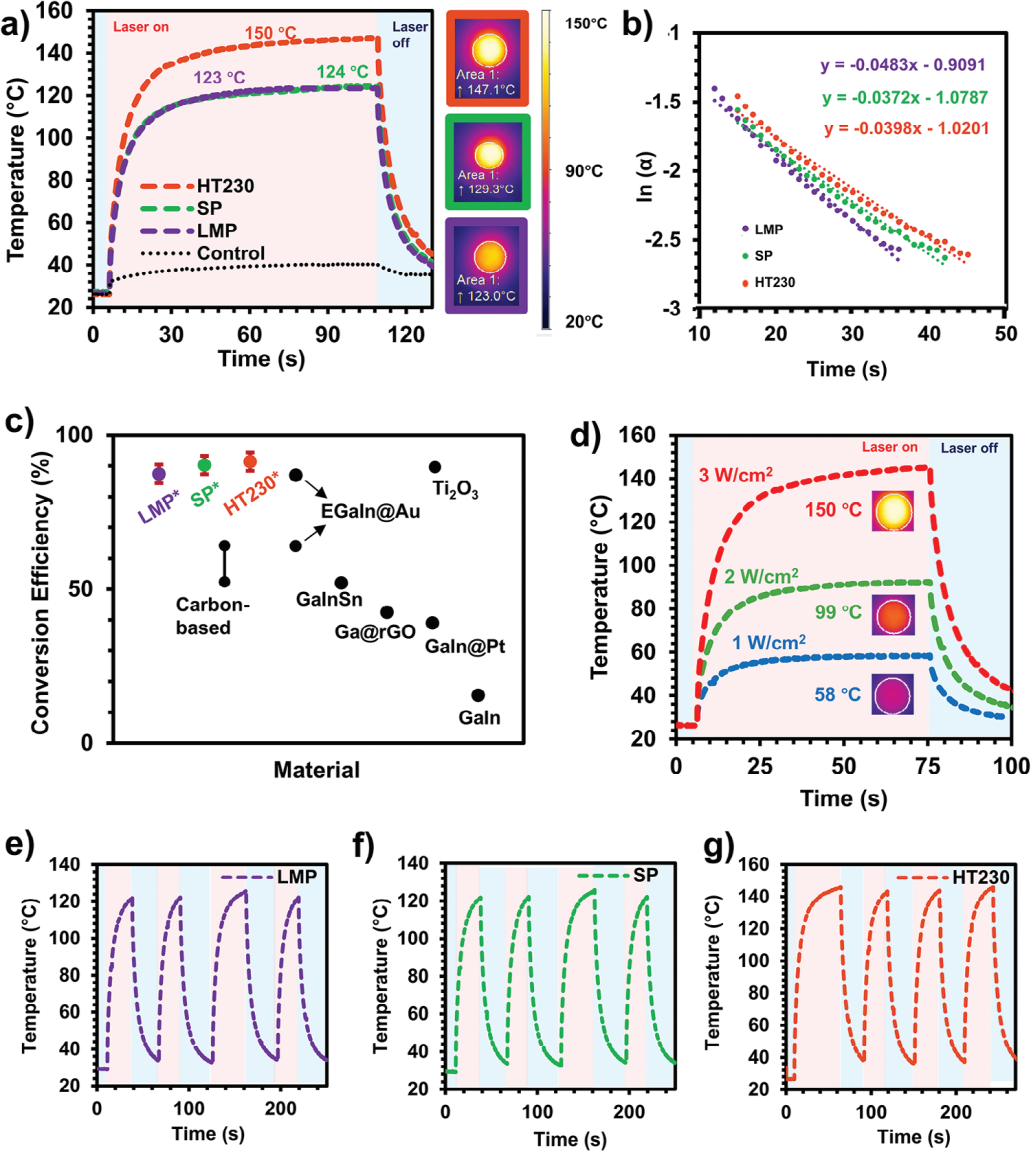
Photothermal characterization of BiSn particle depositions. a) Surface temperature evaluation of HT230, SP, and LMP under the laser and after the irradiation is switched off. The steady‐state temperatures are indicated on the curves. Thermal images showing the maximum steady‐state temperatures within the area of measurement indicated on the photographs with the temperature scale on its right. b) Exponential decay functions of samples representing instantaneous temperature change with respect to concerning ambient temperature obtained from cooling curves. Dashed lines are linear fit equations to the functions. c) Photothermal conversion efficiencies of LMP, SP, and HT230 together with the photothermal conversion efficiencies of Ga‐based liquid metal particle systems (EGaIn@Au.^[^ ^5^, ^42^ ^]^ GaIn, GaInSn, and GaIn@Pt,^[^ ^39^ ^]^ Ga@rGO.^[^ ^41^ ^]^), and conventional photothermal materials (carbon‐based,^[^ ^56^, ^57^ ^]^ and Ti_2_O_3_^[^ ^25^ ^]^), (*) this work. d) Tunability of the surface temperature of the HT230 by adjusting the power density of the laser illumination. The thermal images at steady state are indicated with the temperature value. Cyclic heating/cooling curves of the LMP e), SP f), and HT230 g) irradiated for four cycles.

To measure the PCEs, the samples were irradiated for 100 s, followed by 25‐s‐breaks, using a 915 nm laser with a power of 1.019 W. According to the results provided in **Figure** **2**a, Δ*T* is ≈96.5 ± 2.0, ≈97.1 ± 2.0 °C, and ≈123.8 ± 2.0 for LMP, SP and HT230, respectively. Figure 2a also includes thermal images showing the samples’ steady surface temperatures, where the circular region indicates the illuminated area (indicated as Area 1 in the thermal image), accompanied by the respective surface temperatures. The cooling rates of the samples can be deduced from the slope of the curves fitted to the experimental result's cooling trace (Figure 2b; see Figure S4, Supporting Information for details). As noticed, LMP exhibited the highest cooling rate, followed by SP and HT230, respectively. The relatively slower cooling rate of HT230 may be attributed to the higher oxide content in the particles establishing the deposition. On the contrary, even though the only difference between LMP and SP is the core metal of particles being liquid or solid, their recorded cooling rates were different, indicating that the metallic core indeed impacts the samples’ photothermal performance.

Inserting the experimental results into Equation (1), PCEs were calculated as 87.4 ± 3.0%, 90.3 ± 3.0%, and 91.2 ± 3.0% for LMP, SP, and HT230, respectively (Figure 2c). The significant rise in temperature during irradiation, coupled with a rapid cooling rate, contributed to the highly efficient photothermal performance of BiSn core–shell particles. Compared to others, the highest PCE of HT230 is attributed to samples’ higher light absorption, thus the higher steady‐state surface temperature, stemming from thicker and defect‐rich nano‐oxide shells of heat‐treated particles forming the deposition, regardless of their relatively slow cooling rate. LMP and SP, on the other hand, exhibited resemblance in light absorption (75%) and steady‐state surface temperatures (≈123 and ≈124 °C, respectively), yet there is about a 3% difference in their PCEs. This difference can be attributed to the difference in their metallic cores of particles. The heat transfer coefficients used in Equation (1) were derived using the cooling rates and heat capacities of the samples, and in these calculations, the samples are considered as particle depositions and the glass underneath. Therefore, it is worth noting that particles correspond to only 1.1 wt.% of the samples, yet they were effective enough to create an observable difference in PCEs. Compared to other representative liquid metal photothermal materials, BiSn core–shell particles exhibit the highest PCE, where two Ga‐based systems were reported as 15.5%^[^ ^39^ ^]^ and 65%,^[^ ^5^ ^]^ and modification of Ga‐based liquid metal core–shell systems with noble metal plasmonic nanoparticles enhanced their efficiency by up to 87% (Figure 2c).^[^ ^42^ ^]^ Furthermore, the performance of BiSn core–shell particles either exceeds or rivals the conventional counterparts, such as carbon nanodots with a PCE of 50.0%,^[^ ^57^ ^]^ carbon spheres with a PCE of 65.0%,^[^ ^56^ ^]^ and Ti₂O₃ with a PCE of 90.45%^[^ ^26^ ^]^ under NIR light (Figure 2c).

Expanding on Equation (1), we show that the surface temperature can be remotely controlled by tuning the irradiation power. As exemplified in Figure 2d, HT230 attained steady surface temperatures of ≈58 ± 2, ≈99 ± 2, and ≈150 ± 2 °C by adjusting the power densities to 1, 2, and 3 W cm^−2^, respectively. Similar behavior can also be explored using a variety of BiSn core–shell particles. It is also possible to adjust power density according to the target surface temperature based on the application.

Figure 2e–g show the repeatability of the photothermal response of the particles. Analysis of the temperature‐time curves reveals similar heating and cooling profiles, as well as peak temperatures for LMP (Figure 2e), SP (Figure 2f), and HT230 (Figure 2g) for four cycles of irradiation. Notably, it has been reported in the existing literature that the cyclic heating and cooling events experienced by liquid metal particles can cause a drop in performance.^[^ ^40^, ^58^ ^]^ Yet, LMP, SP, and HT230 utilized in this study exhibited consistent photothermal behavior during four repeated heating and cooling cycles.

### 2.3 The Role of the Core–Shell Structure on the Photothermal Performance

**Figure 3:**
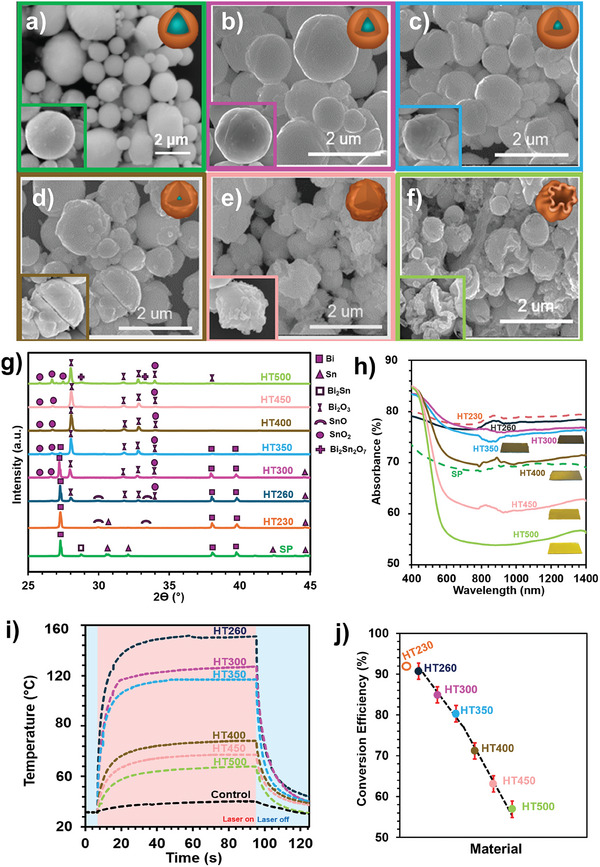
Characterization of heat‐treated (HT) BiSn core–shell particles. SEM micrographs of a) SP prior to heat treatment and HT300‐500 samples at b) 300 °C (HT300), c) 350 °C (HT350), d) 400 °C (HT400), e) 450 °C (HT450), and f) 500 °C (HT500). Note that secondary electron SEMs of SP, HT230, and HT260 are similar. g) XRD patterns of SP and HT230‐500 samples. h) Absorbance spectrum and photographs of HT260‐500 particle depositions, i) surface temperature evaluation of HT260‐500 samples under laser illumination, j) photothermal conversion efficiencies of HT260‐500 samples together with the value of HT230 as a reference.

Non‐noble metals exhibit a notable tendency toward oxidation, where this protective oxide is instrumentalized in the fabrication and applications of liquid metal particles to form core–shell metal‐oxide structures.^[^ ^36^, ^46^, ^59^, ^60^, ^61^, ^62^ ^]^ Our recent study,^[^ ^46^ ^]^ showed that this oxide shell can be engineered not only to form compartmentalized particles but also to obtain thicker oxide shells. In this study, we found that the oxide shell has an impact on the light absorption properties, and a thicker oxide layer leads to greater light absorption and, therefore, a darker color of the particle deposition. Extending this concept, we treated solidified BiSn core–shell particles (SP) at high temperatures in an oxygen‐containing atmosphere and investigated the effect of further changes in core–shell structure on the samples’ photothermal properties (**Figure** **3**).

Figure 3a shows the initial state of the particles of SP. The SEM micrographs of the heat‐treated particle depositions at temperatures 300 °C (HT300), 350 °C (HT350), 400 °C (HT400), 450 °C (HT450), and 500 °C (HT500) are shown in Figure 3b–f, respectively. It is evident that the particles maintained their spherical shape and integrity when treated at 230, 260, or 300 °C (Figure 3a,b). However, particles began to lose their shape at 350 °C (Figure 3c) and even broke into pieces for treatments at 400 and 450 °C (Figure 3d,e). The particle form is barely identifiable when treated at 500 °C (Figure 3f). X‐ray diffraction (XRD) analyses were conducted to identify the changes in the crystal content of these samples. The diffraction patterns in Figure 3g correspond to crystalline Bi (PDF 04‐006‐7762), Sn (PDF 4–673), and crystalline metastable Bi_2_Sn phase (reported in Pilloni et al.^[^ ^63^ ^]^), SnO (PDF 04‐008‐4977), SnO_2_ (ICDS 262767), Bi_2_O_3_ (ICDS 260800), and Bi_2_Sn_2_O_7_ (ICDS 86845). SP showed distinct crystalline peaks for metallic Bi, Sn, and Bi_2_Sn. Upon treating SP at 230 °C (HT230) and 260 °C (HT260), the SnO phase was also observed. The HT260 sample contains both SnO and SnO_2_ phases. For the HT300‐350 samples, metallic Bi and Sn peaks were still identifiable, along with the emergence of Bi_2_O_3_ and SnO_2_. The presence of cracks and deviations from the spherical shape can be associated with the expansion stemming from the increase in the oxide content (Figure 3d–f). Metallic Bi and Sn peaks almost completely disappeared upon treatment at 400 or 450 °C. Therefore, we can conclude that the metal core can be protected at a certain level up to 400 °C. When particles were treated at 500 °C, a mixed oxide phase, Bi_2_Sn_2_O_7_, was also formed, and the integrity of the particles was almost completely lost.

The absorption spectrum of the HT samples is provided in Figure 3h, along with corresponding photographs of the particle depositions. The HT260, HT300, and HT350 exhibit continuous (flat) broadband absorption characteristics similar to those of SP and HT230. However, upon treatment at 450 °C, the NIR absorption of the samples gradually decreased, coinciding with the formation of a sharper visible absorption edge ≈580 nm (Figure S6, Supporting Information), resembling the typical optical absorption characteristics of Bi_2_O_3_.^[^ ^64^ ^]^ The photographs of the HT particle depositions displayed a noticeable color change from dark gray to yellow, suggesting the presence of Bi‐oxides.^[^ ^64^, ^65^ ^]^ This color change in the coatings corresponded to the transition from the dominance of metallic phases to oxide phases and confirmed the formation of an absorption edge. The oxide‐like absorption behavior observed for 450 and 500 °C heat‐treated samples aligned with the formation of oxide phases at the expense of metallic ones, as well as the distortion of core–shell integrity observed in SEM micrographs (Figure 3d–f). These results proved that the metal core‐oxide shell structure was pivotal for broadband light absorption. Moreover, the proportion and the content of the oxide shell layer were found to have influenced the level of optical absorption that could be achieved. Even though heat treatment at 230 °C enhanced broadband absorption above 80% (Figure 3h), the treatments at higher temperatures were not as effective. The NIR absorbance (780–1400 nm) of HT260 was slightly lower than that of HT230 (≈80%); for HT300 and HT350, it was ≈75%, which decreased to 70% for HT400, and further declined to 55% for the HT500. SnO_2_ and Bi_2_O_3_ are moderate‐bandgap semiconductor metal oxides with a lower defect tendency to nanoscale SnO; thus, altering the oxide content may lead to a decrease in light absorption.^[^ ^36^, ^50^ ^]^ As a result, the core–shell structure of particles establishing HT230 was optimal for achieving maximum broadband light absorption capability.

Although there was a decrease in the broadband absorption capacity due to oxidation, the photothermal properties of the HT samples were still observed. The surface temperatures of the particle depositions under the NIR laser were measured, and the results (Figure 3i) showed that the HT260 reached ≈150 °C, HT300 reached ≈125 °C, and the HT350 reached almost ≈120 °C, similar to the SP (Figure 2). On the other hand, the HT400 showed a significant drop in the photothermal performance, and its surface reached only ≈65 °C. In comparison, the maximum surface temperatures of HT450 and HT500 samples gradually decreased to ≈50 and ≈45 °C, respectively. The concurrent distortion of particle integrity, diminishing metallic content, and reduction in NIR absorption observed in the HT400 and HT500 resulted in a significant deterioration in their photothermal performance.

The conversion efficiency values (Figure 3j) followed the trend of maximum temperature values attained by the samples. The efficiency of HT samples were: ≈90.7 ± 2%, 84.9 ± 2%, 80.3 ± 2%, 71.2 ± 2%, 63.1 ± 2%, and 59.9 ± 2% for HT260, HT300, HT350, HT400, HT450 and HT500, respectively. Details of the calculations are available in Supporting Information. Note that both light absorption, maximum surface temperature, and conversion efficiency of HT260 are similar to that of HT230, both containing SnO phase on their shells detectable by XRD. However, with the decreasing SnO content, the efficiency dropped. The disappearance of the metal core in samples treated at 400 °C (HT400) and above resulted in a significant reduction in all photothermal performance metrics, including the light absorption capacity, maximum temperatures achieved, and the PCE value. It is worth emphasizing even though the metallic content decreased to a level where it is barely detectable when heat treatment was employed at 350 °C (HT350), the performance drop occurred for HT400 rather than HT350, as observable from the dramatic change in the slope of the plot presented in Figure 3j. This observation supports the hypothesis that the core–shell structure is vital for good photothermal performance.

Combining the results of Figures 2 and 3, the presence of defect‐rich SnO and metallic phases, and the metal core‐oxide shell structure are all influential in the remarkable photothermal performance of BiSn particles. In defect‐rich oxides, the vacancies act as phonon scattering points and may increase the vibrations due to light absorption, leading to broadband light absorption and photothermal conversion, respectively. The amount and the chemistry of the oxide on the shell, which are tunable in liquid metal‐derived particles, are found effective in both the level and the behavior of the light absorption. The presence of metal cores influenced the maximum achievable temperatures and/or the heating and cooling rates of the samples, probably because of their relatively high thermal conductivity and low heat capacity. The ratio of oxide to metal phases and the core–shell structure seems to affect the light absorption capability, the surface temperatures achieved, and the heating/cooling rates, thus the PCE. Moreover, the interface of the metal core and the oxide shell may lead to multiple light scatterings,^[^ ^66^ ^]^ leading to broadband light absorption and thus enhancing the PCE.

### 2.4 Solar Water Evaporation System

**Figure 4:**
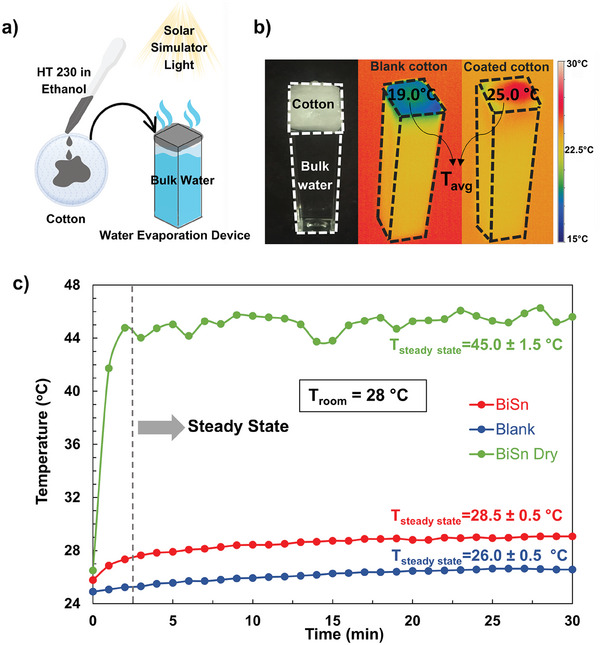
a) Schematic illustration for the solar absorber layer (left) and the solar evaporation measurement setup (right). b) Photograph of the setup prior to the experiment, along with thermal images capturing the system with particle‐coated cotton and the blank cotton recorded after reaching the steady state (5 minutes after the light exposure). c) Time‐dependent temperature changes recorded using a thermocouple under the solar simulator for blank (blue) and BiSn particles coated samples (green and red are for dry and wet states).

In the context of interfacial water evaporation systems,^[^ ^67^, ^68^ ^]^ our research explored the efficacy of BiSn core–shell particles due to their broadband light absorption capabilities. We designed a simple solar water evaporation device, as illustrated in **Figure** **4**a, without specific heat loss precautions. This setup utilized water‐absorbent cotton as both substrate and water transport medium, with heat‐treated BiSn particles at 230 °C (forming HT230) chosen for their relatively higher solar‐thermal conversion efficiency (Figure 2c).

Initial tests using a NIR laser to heat the particle‐coated cotton resulted in combustion due to excessive surface temperatures. Subsequent experiments conducted under a solar simulator with 1000 W m^−2^ intensity (1 Sun, spectral irradiance of which is given in Figure S7, Supporting Information) were more successful and practically more relevant for this specific application. Thermal imaging (Figure 4b) showed a noticeable temperature increase in the particle‐coated cotton compared to the untreated sample. This led to a higher water evaporation rate of 1.9 kg m^−^^2^ h—double the rate observed under dark conditions. This net solar evaporation rate difference of 1.0 kg m^−^^2^ h recorded by the BiSn particle‐coated cotton system is significantly higher than the control sample of plain cotton, which exhibited only a 0.4 kg m^−^^2^ h evaporation rate. Details of the evaporation rate calculations are available in Supporting Information.

While the average surface temperature of the particle‐coated dry samples was 45.0 ± 1.5 °C, it decreased to 28.5 ± 0.5 °C when the same cotton was wet, i.e., when placed on top of the water‐filled tube (Figure 4c), suggesting that efficient heat utilization conducive primarily for evaporation of water rather than heating the bulk water. Please note that the surface temperature of the particle‐coated cotton was still higher than the blank one, even under wet conditions. Despite the simplistic configuration of the setup, the solar‐to‐water vapor conversion efficiency could reach 68% using BiSn core–shell particles.

These results demonstrate the potential of BiSn core–shell particles in solar water evaporation applications, offering a cost‐effective and scalable alternative to traditional carbon‐based materials.^[^ ^19^, ^69^, ^70^ ^]^ Future optimizations of the device design are expected to further enhance evaporation performance.^[^ ^71^ ^]^

### 2.5 NIR‐Sensitive PDMS/BiSn Photo‐Actuator

**Figure 5:**
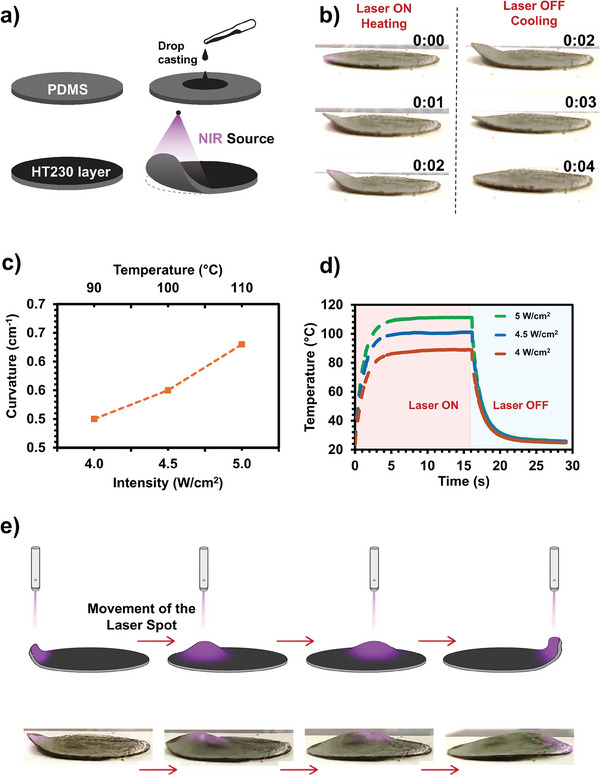
a) Schematic representation of the fabrication route (top) and actuation mechanism (bottom) of the photo‐actuator. b) Time frames (captured at 1‐second intervals) showing the actuator heating under NIR laser irradiation (left) and cooling when the laser is turned off (right). c) Maximum curvature is attained by the actuator at the given light intensity and corresponding temperature. d) Heating and cooling curves of the actuator for different light intensities e) Schematic demonstrating dynamic shape adaptation by moving the laser source (top). The digital photographs of the actual sample (bottom). The purple circles indicate the illumination areas of the laser.

While solar evaporation benefits from materials that absorb a wide range of light, sensory applications require materials with exceptional photothermal conversion efficiency, characterized by rapid heating and cooling rates.^[^ ^9^, ^11^, ^72^ ^]^ As we prove here that BiSn core–shell particles stand out as effective advanced photothermal material due to their high conversion efficiency and rapid cooling capabilities, we aim to demonstrate this efficacy in a new NIR‐sensitive bilayer photo actuator. This proof‐of‐concept study introduces a NIR‐sensitive bilayer soft photo actuator consisting of a high thermal expansion layer made from PDMS and a low thermal expansion layer embedded with photoactive BiSn core–shell particles (forming HT230). The actuator was fabricated by drop‐casting the particles suspended in ethanol onto a PDMS (170 µm thick) substrate, followed by ethanol evaporation to form the NIR‐sensitive layer in one step (**Figure** **5**a).

Upon irradiation with 915 nm NIR light at intensities ranging from 4 to 5 W cm^−^^2^, the actuator exhibited rapid responsiveness, achieving maximum curvature within 2 s and returning to its original state in another 2 s when illuminated at the light intensity of 5 W cm^−^^2^ (Figure 5b; Video S1, Supporting Information). Subsequent experiments at varying light intensities revealed that the actuator's curvature increased proportionally with light intensity, reaching a peak curvature of 0.63 cm⁻¹ at 5 W cm^−^^2^, corresponding to a surface temperature of 110 °C (Figure 5c). These results indicate that light intensity directly modulates the temperature and the actuator's curvature. The heating and cooling profiles were consistent across different intensities, with the actuator rapidly reaching equilibrium temperatures and cooling uniformly (Figure 5d).

The actuator's ability to dynamically adapt shape was further demonstrated by moving the laser spot across its surface, inducing rapid and local transformations (Figure 5e; Video S2, Supporting Information). This adaptability underscores its potential for various applications requiring dynamic responsiveness.

Table S1 comprehensively compares the performance metrics of various NIR‐responsive bimorph photothermal soft actuators. PDMS/BiSn photo‐actuator distinguishes itself through its straightforward production process, rapid response and recovery rates, and high equilibrium temperatures without compromising curvature under varying NIR intensities. Despite being the thickest among the compared actuators, the PDMS/BiSn photo‐actuator achieves one of the most rapid response times, the highest temperature difference, and a comparable curvature value.

## 3 Conclusions

This study introduced liquid metal‐derived BiSn core–shell particles as novel materials for broad‐spectrum light absorption and efficient photothermal conversion. Our experimental findings revealed that these particles achieved continuous absorption levels exceeding 75% across the spectral range of 400 to 1400 nm, with certain treatments reaching up to 80%. Under NIR laser illumination, the BiSn particle depositions demonstrated impressive photothermal properties, heating up to 150 °C, with photothermal conversion efficiencies reaching as high as 91.2%, the highest reported among liquid metal particle systems in the literature.

A novel approach in this study was treating liquid metal particles as core–shell hybrid structures of metal and oxide to investigate their photothermal properties. This perspective revealed the significant enhancement in the particles' photothermal properties and demonstrated the critical role of altering core and shell structures. Combining a thermally conductive metal core with a defect‐rich oxide shell, the unique particle structure was pivotal in achieving these enhanced properties, allowing for the facile synthesis of particles with optimal core–shell characteristics. Additionally, the performance of the BiSn core–shell particles in advanced proof‐of‐concept studies was remarkable, showing their potential as a solar absorber layer for water evaporation and an actuation‐trigger layer for sensory actuators.

All in all, the unique core–shell structure of the particles, achieved via the liquid metal template, was crucial in attaining these results. Our findings not only showcased the potential of BiSn core–shell particles but also paved the way for further research and development in advanced photothermal materials, indicating significant opportunities for future innovations with tailored photothermal properties.

## 4 Experimental Section

### Particle Deposition on Glass Substrate

The eutectic BiSn liquid metal core–shell particles were produced from a eutectic BiSn alloy (58 wt.% Bi) via the droplet‐emulsion technique—the solidification‐controlled compartmentalization procedure described by Ulusel et al.^[^ ^46^ ^]^ was followed for the modification leading to the formation of the solidified and the heat‐treated particles.

Particle depositions were prepared by drop‐casting liquid metal particle dispersions in ethanol onto preheated (70 °C) glass slides (≈1 mm‐thick, Isolab Cover Glass). The process continued until a visually uniform gray color was obtained (32.8 g m^−2^), roughly indicating the coverage of the substrate's surface by particles. Therefore, the LMP (BiSn liquid metal particle deposited layers on glass) was prepared. Then, the LMP was cooled down to −20 °C (described as a lamellar particle synthesis procedure in Ulusel et al.’s study), and solidified particle deposited layers on glass (SP) were obtained. SP was heat treated at 230 °C for 6 h to obtain the deposition on glass, which was made up of layers of heat‐treated particles at 230 °C (HT230) (described as the striped particle synthesis procedure in Ulusel et al.’s study).

SP samples were also treated at elevated temperatures to obtain different oxide shell thicknesses. Six SP samples were subjected to heat treatment at 260, 300, 350, 400, 450, and 500 °C for an hour in a muffle furnace (Mikrotest) in the open air to produce heat‐treated particle depositions. Samples were labeled HT260, HT300, HT350, HT400, HT450, and HT500 according to the heat treatment temperature. The heating rate was 2 °C s^−1^.

### Materials Characterization

The particle depositions’ integrity, the thickness of the layers, and the deposited particles were analyzed using FE‐SEM (FEI Nova NanoSEM 430). The deposited layer thicknesses were measured from the SEM micrographs using the instrument's software. Five measurements were taken from multiple regions, and three different samples were utilized for the measurements. The heat‐treated samples were coated with a thin layer of gold for analysis using an Emitech SC7620 Sputter Coater. TEM analyses were done using FEI Talos F200S using platinum‐coated particles. In TEM analyses, particles smaller than 100 nm were used; to obtain them, the particles were centrifuged for 15 min at 5000 rpm, and the supernatant fluid was drop‐coated on carbon‐coated copper grids and left to be dried.

Crystallographic analyses of the particles were performed by X‐ray diffraction (XRD, Bruker D8 Advance) using Cu‐Kα radiation (0.154 nm) operating between 20° and 45° at a scanning speed of 2°min^−1^.

The optical properties of the samples were characterized using a UV–Vis–NIR spectrophotometer (Shimadzu UV‐2600i). An integrating sphere (Shimadzu ISR‐2600Plus) was used to collect the total reflectance and transmittance spectra between 200 and 1400 nm wavelengths. The absorbance values were then calculated by subtracting the sum of the total reflectance and transmittance values from 100%. The optical properties of the glass substrate are available in Figure S8 (Supporting Information). Due to the inherent light absorption of the glass substrate, values below 400 nm were excluded from the optical analysis.

The photothermal properties of the samples were measured using a thermal camera (Optris PI 640i). In contrast, the samples were simultaneously illuminated from the top with an Nd: YAG laser (Thorlabs, illuminating at a wavelength of 915 nm and a laser intensity of 3 W cm^2^ unless otherwise stated). The samples were placed on a porcelain combustion boat (Isolab) to minimize thermal loss by conduction. A bare glass substrate (Isolab Cover Glass) was the control sample. The emissivity of the surfaces was determined by equilibrating the surface of the samples and ambient temperature in the experimental environment before laser illumination. The measurements were repeated three times. The interaction volume accepted for the photothermal calculations is illustrated in Figure S3 (Supporting Information).

Particle size distribution was measured by Malvern Zetasizer Ultra instrument in single angle mode (Backscatter, 173°). The 0.1 g of particles were dispersed in 20 ml ethanol for measurement. The samples were evaluated three times, and the average results were reported. Zeta potential measurements were conducted using the same suspensions and the same device.

### Solar Water Evaporation System

The solar evaporation system used in this study consisted of a cotton layer that served as a light absorber and a soaking layer for water. To produce the cotton layer, a rectangular area of 1.3 cm^2^ was cut from the round cotton disc (Watsons Disc Cotton). BiSn core–shell particles heat treated at 230 °C suspended in ethanol were deposited onto the cotton surface via drop‐casting. The deposition was continued until a uniform gray color on the cotton surface was obtained, corresponding to a particle loading of 0.02 g. The coated cotton was placed on top of a 10 × 10 × 45 mm polystyrol/polystyrene tube (SARSTEDT AG & Co. KG) after filling it with deionized water (DIW). As a control, an identical setup was constructed using blank cotton. The solar evaporation performance of the system was measured using a solar simulator (Ossila LED‐Based Class AAA Solar Simulator, whose spectral irradiance is provided in Figure S7, Supporting Information) operating at 1 sun (1000 W m^−2^). The distance between the simulator and the sample was set to 8.5 cm, as specified for this instrument. The temperature of the cotton lying on the upper surface of the system was recorded using a K‐type thermocouple (Elimko) connected to a data logger (Ordel). Temperature values were recorded for 30 min. Each measurement was repeated three times, and the average was plotted. The temperature changes were also recorded using a thermal camera (Optris PI 640i).

The solar evaporation rate of the device during illumination was measured by weighing the device on an electronic balance immediately before and after its exposure to light. The details of the calculations are provided in the Supporting Information.

### NIR‐Sensitive PDMS/BiSn Photo‐Actuator

The NIR‐sensitive PDMS/BiSn photo‐actuator was fabricated so that the heating layer acted as a low thermal expansion layer. To do so, a silicone elastomer PDMS (Sylgard 184) was mixed with the curing agent at a ratio of 10:1 and laid on glass through doctor blading with a wet thickness of 200 µm (thickness was measured as 170 µm after curing). The mixture was then cured on glass at 80 °C for 45 min in an oven. Following curing, the 6‐mm‐diameter PDMS samples were punched to obtain a high‐thermal‐expansion layer. The punched samples were drop‐coated with 0.2 g of BiSn particles heat treated at 230 °C suspended in 10 mL ethanol. The coating process was continued until a uniform gray color (32.8 g m^−2^) was obtained on the PDMS surface. The coated BiSn particle layer acted both as a low thermal expansion layer and a photoactive heating layer for actuation.

The actuation event under laser illumination was recorded using the actuator placed on a porcelain combustion boat (Isolab) to avoid thermal losses beneath the Nd: YAG laser source adjusted to irradiate at 915 nm. The PDMS/BiSn photo‐actuator was illuminated with different power densities (4.0, 4.5, and 5.0 W cm^−2^). During illumination, the temperature was measured via a thermal camera (Optris PI 640i) simultaneously with the actuation, and a video was recorded from the side of the actuator to observe the bending motion of the actuator. The power densities were arranged such that only a portion of the actuator was irradiated by the laser spot.

## Conflict of Interest

The authors declare no conflict of interest.

## Supporting information



Supporting Information

Supplemental Video 1

Supplemental Video 2

## Data Availability

The data that support the findings of this study are available from the corresponding author upon reasonable request.
