# Supplementary material for: Bismuth‐Tin Core–Shell Particles From Liquid Metals: A Novel, Highly Efficient Photothermal Material that Combines Broadband Light Absorption with Effective Light‐to‐Heat Conversion
Source: Adv Sci (Weinh). 2024 Oct 7;11(45):2407771. doi: 10.1002/advs.202407771 (PMC11615822; doi:10.1002/advs.202407771)
Supplement: Supplementary file 1 — Supporting Information [file ADVS-11-2407771-s003.docx]

Supporting information for:

**Bismuth-Tin Core-Shell Particles from Liquid Metals: A Novel, Highly Efficient Photothermal Material that Combines Broadband Light Absorption with Effective Light-to-Heat Conversion**

Dogu Seyda*^1^*, Orcun Dincer^1,^*^2^*, Duygu İnce*^1^*, Murathan Cugunlular*^1^*, Husnu Emrah Unalan*^1^*, and Simge Çınar-Aygün*^1*^*

*^1^Dept. of Metallurgical and Materials Engineering, Middle East Technical University (METU), 06800 Ankara, Türkiye*

*^2^* Dept. of Chemical and Materials Engineering, Concordia University, Montreal, Quebec, Canada

**Corresponding author, email: csimge@metu.edu.tr; ^2^ Present address*


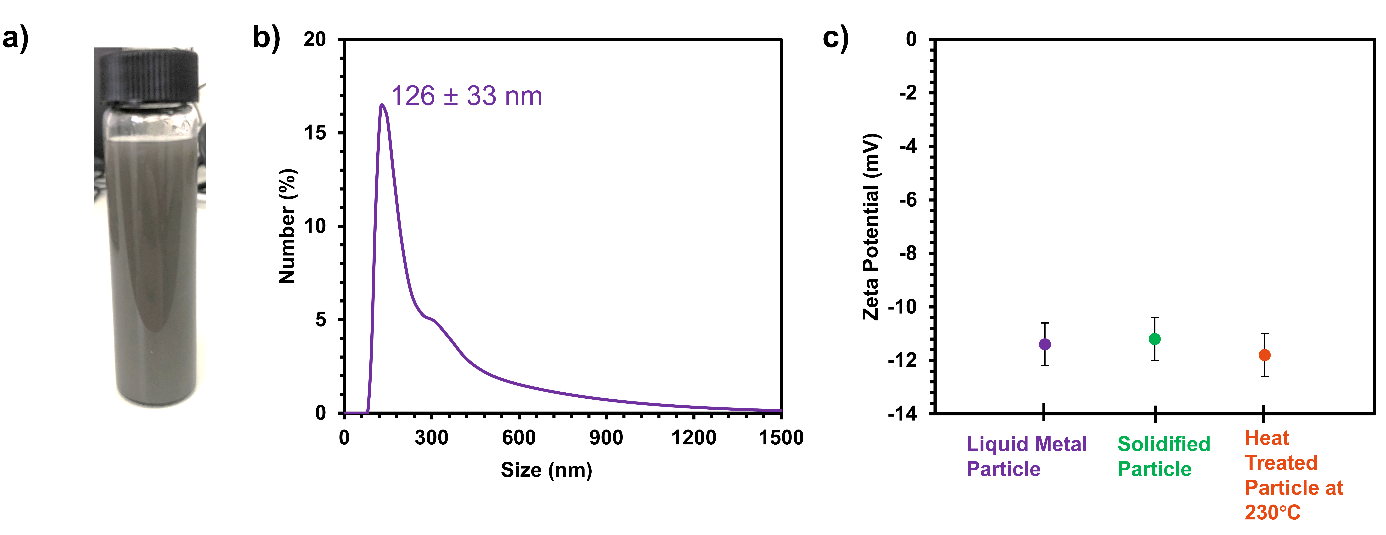


**Figure S1.** *Colloidal characteristics of BiSn core-shell particles in suspension prior to drop-casting a) Digital photo showing stability of BiSn liquid metal particles in ethanol. The particles stayed suspended in ethanol for at least one week. b) Particle size distribution of BiSn liquid metal particles in ethanol. The number weighted average of particle size is 126 ± 33 nm c) Zeta potential determination of BiSn liquid metal particles in ethanol. liquid metal particles, solidified particles and heat-treated particles at 230 °C have nonzero zeta potential values in ethanol that keeps them suspended during drop-coating process.*


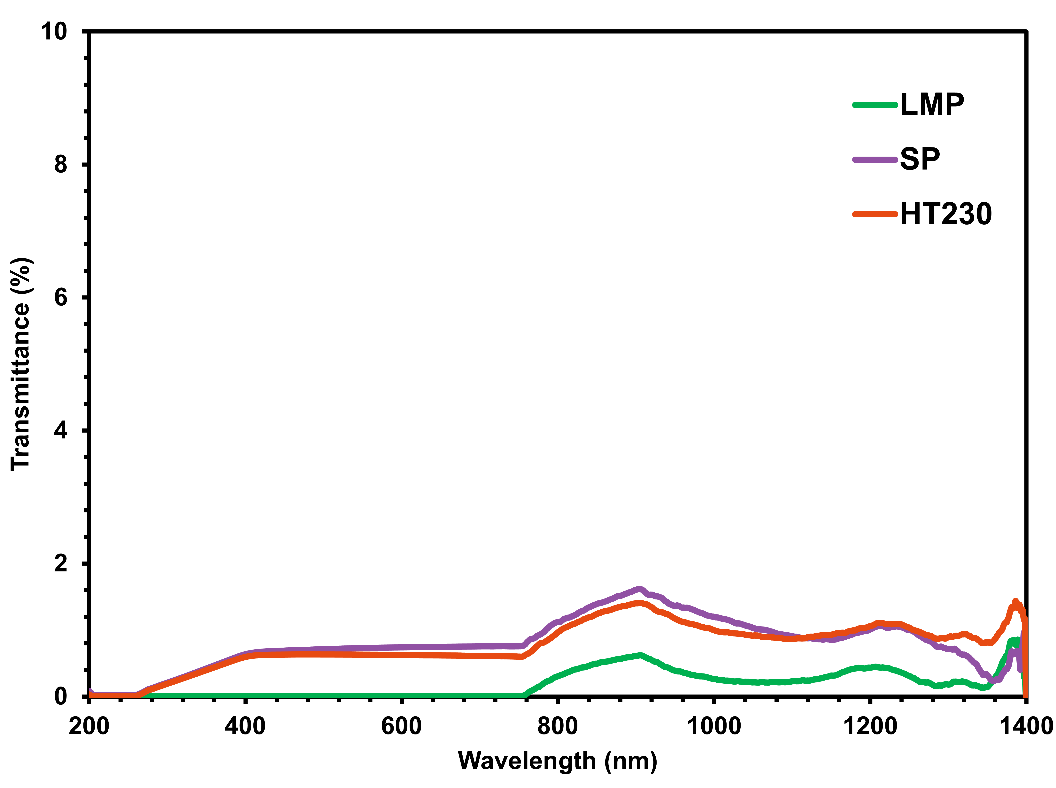


**Figure S2.** *Total transmittance (%) spectrum of LMP, SP and HT230.*

**Photothermal conversion efficiency calculations**

The light-to-heat external conversion efficiencies of BiSn core-shell particles were calculated via a modified photo-calorimetric method as described in Cai et al.^[1]^

The efficiency calculations of the system were done assuming that the laser-particle interactions are restricted to the volume described as the following. The circular laser spot size is 6000 μm in diameter, particle deposition has 30 ± 5 μm thickness and the glass substrate beneath the particles has 1000 ± 20 μm thickness. It is assumed that the heating/cooling events due to the photothermal effect from the laser-illuminated particle deposition at the top occur only in the described interaction volume as schematized in Figure S3.

| **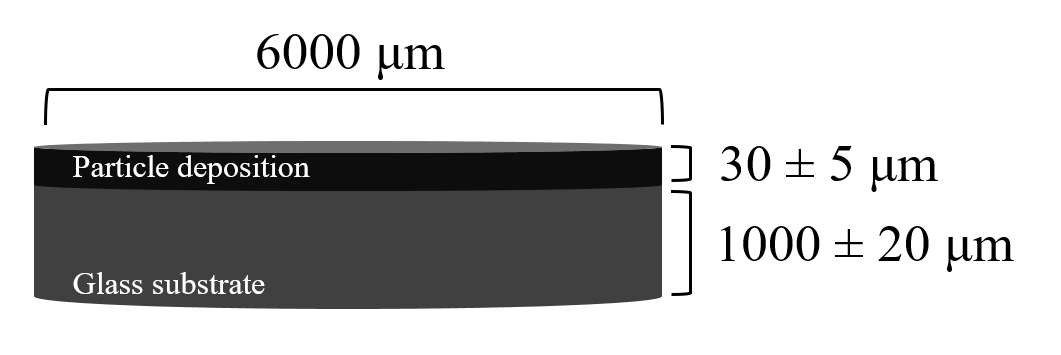** |
| --- |
| **Figure S3.** The schematic shows the interaction volume of photothermal event. |

The efficiency *η*, is defined as the ratio of heat generation (*Q_gen_*) to irradiance energy of the light source *(Q_irr_*) given in Equation S1.

| $\eta=\frac{Q_{gen}}{Q_{irr}}$ | (Equation S1) |
| --- | --- |

Equation S1 is applied for illumination from a 915 nm laser at a power density of 3 W/cm^2^. Under light source, the temperature of the particles is expected to show an increase initiating from room temperature (*T_env_*) to maximum steady-state surface temperature (*T_max_*) monitored by a thermal camera. In experiments, the temperature differences achieved after illumination were measured as ~96.5 ± 2.0 °C for LMP, ~97.1 ± 2.0 °C for SP, and ~123.8 ± 2.0 °C for HT230. Based on the conservation of energy, the heat generated due to the photothermal effect of the particles should be equal to the heat loss to the surroundings (Equation S2).

| $Q_{gen}=Q_{loss}=h \times s \times(T_{max}-T_{env})$ | (Equation S2) |
| --- | --- |

In Equation S2, *h* refers to the heat transfer coefficient, and *s* is the surface area perpendicular to the illumination. At a steady state, this equation should be valid as the temperature difference between the surface and the environment stays the same. After the light source is switched off, the surface cools down, and the time dependence of instant temperature (*T*) drop can be interpreted by Equation S3.

| $T=T_{env}+(T_{max}-T_{env})exp(-Kt)$ | (Equation S3) |
| --- | --- |

In Equation S3, *t* is the time. The values of *K* (the slope) were found by fitting the particles’ cooling curves as given in Figure S4, where $\frac{T-T_{env}}{T_{max}-T_{env}}$ is defined as α. From the slope of the fitted curves, the *K* values for the particles were determined as 0.0483, 0.0372, and 0.0398 s^-1^ for LMP, SP, and HT230, respectively. K value, then, is defined by Equation S4.

| $K=\frac{hs}{\Sigma_{i}mc_{p}}$ | (Equation S4) |
| --- | --- |

In our experiments, *m* is the mass of the particles (0.92 ± 0.01 mg) and the glass (80 ± 10 mg). The heat capacities, *c_p_*, of the solidified BiSn particles and heat treated BiSn particles at 230° are 0.167 J/g⋅K, and 0.201 J/g⋅K for BiSn liquid metal particles (CAS #: 12010-55-8, American Elements), and for the glass substrate, it is 0.870 J/g⋅K ^[2]^. The composition of the liquid metal particles and solidified particles were assumed to be eutectic composition and heat capacity values were calculated accordingly. For LMP, the heat capacity value in the liquid state was used, and the difference between the heat capacity values of undercooled (metastable) and equilibrium liquid states was neglected. Then, from Equation S4, $hs$ values for the coatings were obtained as 6.92, 7.11, and 6.01 J⋅K^-1^⋅s^-1^ for LMP, SP, and HT230.

| 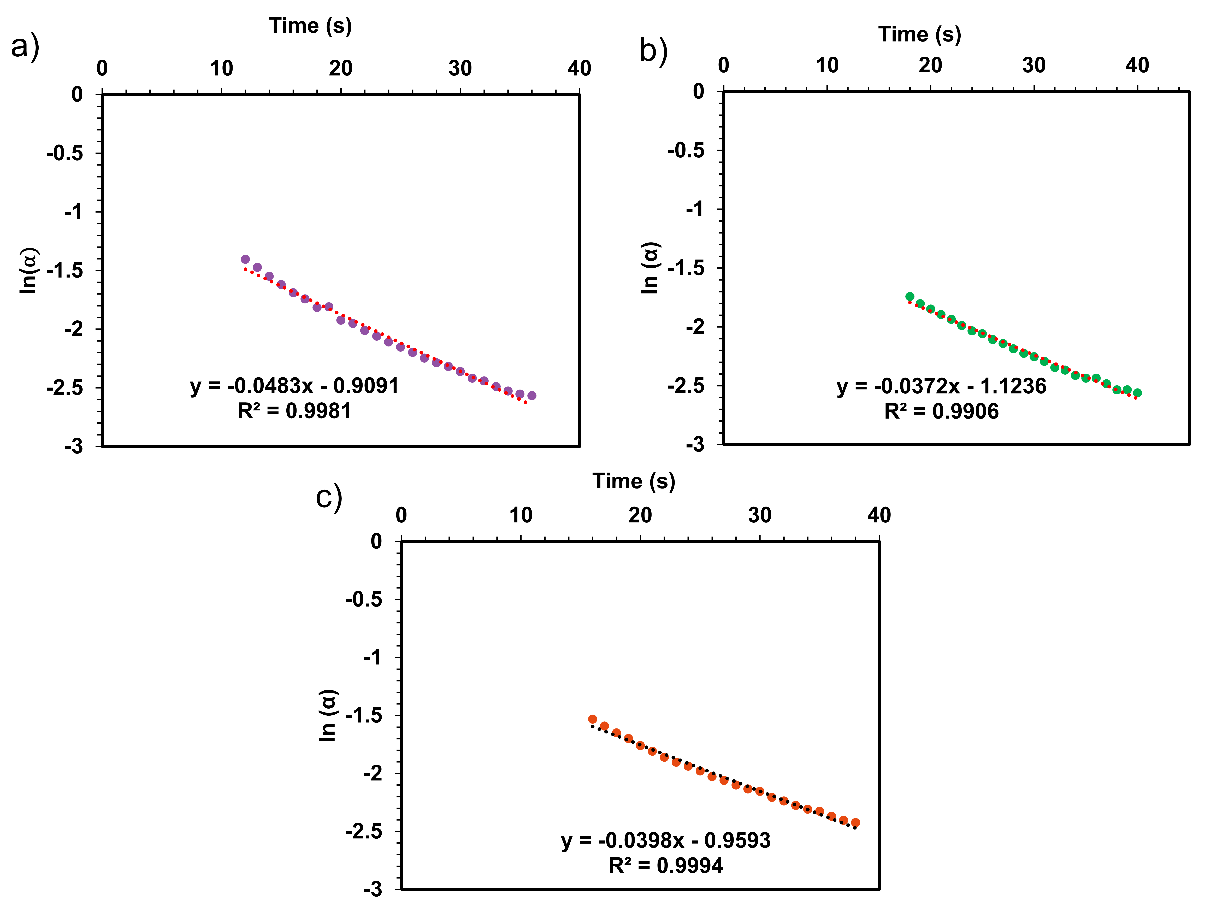 |
| --- |
| **Figure S4.** *Cooling curves showing the change of ln (α) with respect to time together with linear fitting lines of a) LMP, b) SP, and c) HT230 under 915 nm laser irradiation. The slope of the fitted lines represents the value of K.* |

According to the above calculations, the external conversion efficiency can be interpreted from Equation S5.

| $\eta=\frac{hs\Delta T}{IA_{\lambda}}$ | (Equation S5) |
| --- | --- |

In Equation S5, *I* is the power (1.019 W), A_λ_ is the absorbance and the normalized absorbances of the films at 915 nm, which are 0.75 for LMP and SP and 0.80 for HT230, respectively, as presented in Figure 1f, were used in calculations. Hence, the photothermal conversion efficiencies of the films were calculated as 87.4 ± 3%, 90.3 ± 3%, and 91.2 ± 3%, for LMP, SP and HT230, respectively. Percent errors were calculated by the following formula: $\frac{(Estimated Result-Actual Result)}{Actual Result}x100$. The results varied based on variances of the mass, thickness and temperature measurements. The reported conversion efficiencies are the medium values. Maximum estimation for the efficiencies is 90.2%, 92.3%, and 94.1% for LMP, SP and HT230, respectively.

The same procedure described above was applied for calculations of the conversion efficiency of heat-treated (HT) samples except their *c_p_* were taken as the maximum and the minimum of the range 0.113 J/g⋅K (Bi_2_O_3_ CAS 1304-76-3), and 0.167 J/g⋅K (eutectic BiSn alloy, CAS 12010-55-8). Cooling curves of HT260-500 samples were given in Figure S5. According to the calculations, the photothermal conversion efficiencies of HT260-500 samples were calculated as ~90.7 ± 2%, 84.9 ± 2%, 80.3 ± 2%, 71.2 ± 2%, 63.1 ± 2%, and 59.9 ± 2% for HT260, HT300, HT350, HT400, HT450 and HT500, respectively. The results varied due to uncertainty of mass, thickness, temperature and heat capacity measurements. The reported values are medium values.

| 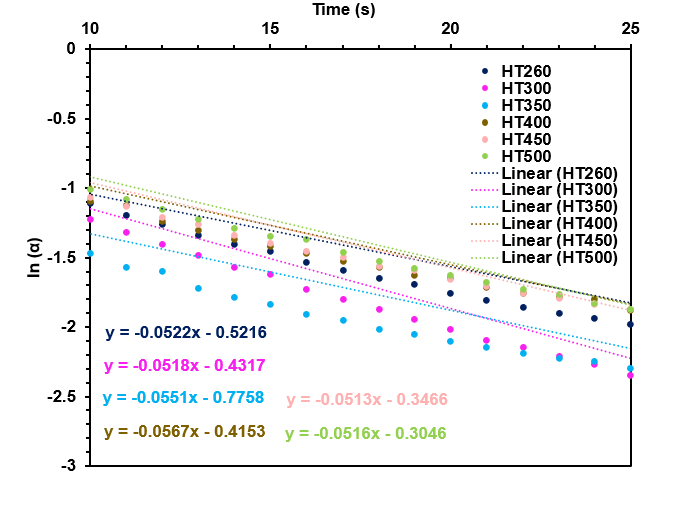 |
| --- |
| **Figure S5.** *Cooling curves showing the change of ln (α) with respect to time together with linear fitting lines of HT260-500 under 915 nm laser irradiation. The slope of the fitted lines represents the value of K.* |


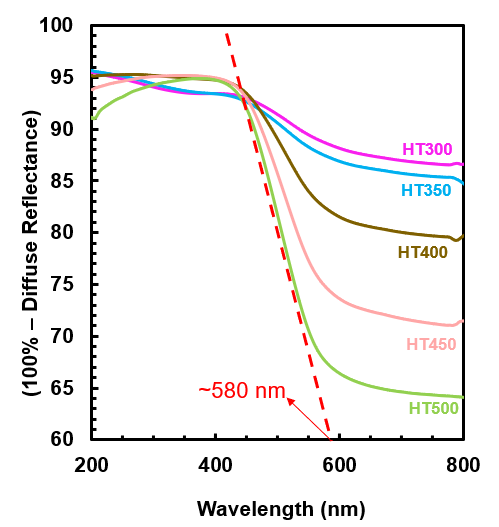


**Figure S6.** *Absorbance spectra of heat treated (HT) particle depositions calculated from their diffuse reflectance values. Baseline corrected with respect to visible light absorber black reference plate. Dashed line indicating the optical absorption edge of HT500.*

**
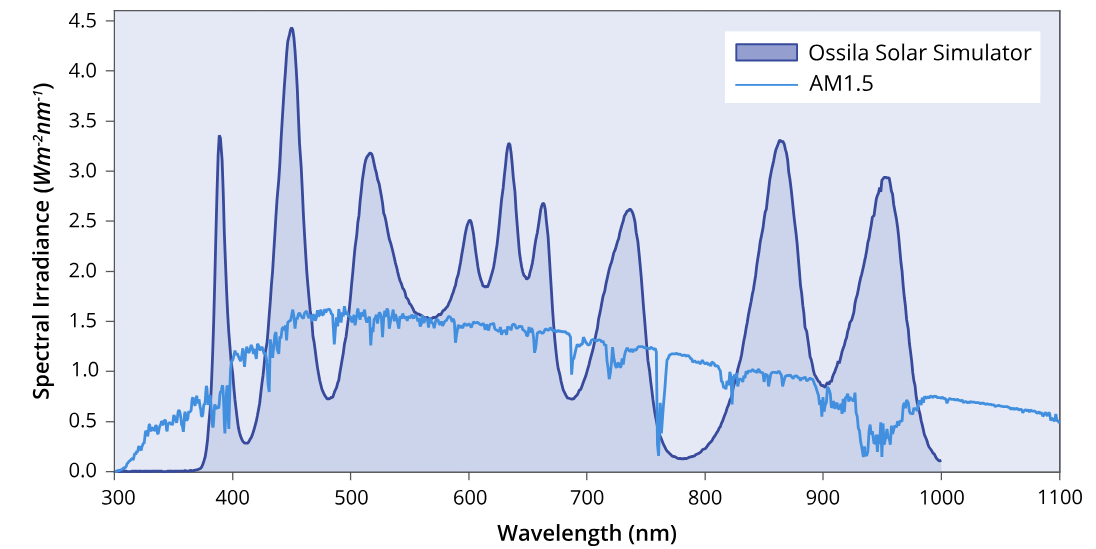
**

**Figure S7.** *Spectral irradiance of the solar simulator (measured at 1 sun intensity, 1000 W/m^2^) together with Reference Air Mass 1.5 Spectra (AM 1.5)* ^[3]^

**Solar Evaporation Rate Calculations**

The solar evaporation rate (*dm/dt*) is calculated using Equation S6, where *Δm* is the mass difference indicating the amount of water evaporated over time, *A* is the area of evaporation surface and *Δt* is the duration of the experiment.

| $\frac{dm}{dt}=\frac{\Delta m}{A\Delta t}$ | (Equation S6) |
| --- | --- |

The surface area of the cotton used as a solar absorber in this study was 132 mm^2^. For the system with BiSn particle-coated cotton, the mass difference recorded in 30 min is equal to 0.127 g under the solar simulator. The mass difference for the same system waiting in the dark for the same periods of time is 0.061 g.

The overall evaporation rate difference created by the system *(dm/dt)_system_* is found by subtracting the mass difference calculated in the dark *(dm/dt)_dark_* from that under the light source *(dm/dt)_solar simulator_* as shown in Equation S7. These values are calculated as 1.92 kg/m^2^×h and 0.92 kg/m^2^×h under a solar simulator and in dark conditions, respectively. Therefore, the net solar evaporation rate difference of BiSn particle-coated cotton system is calculated as 1.0 kg/m^2^×h.

| $\left( \frac{dm}{dt} \right)_{system}=\left( \frac{dm}{dt} \right)_{solar simulator}-\left( \frac{dm}{dt} \right)_{dark}$ | (Equation S7) |
| --- | --- |

The same calculations are computed for the set-up with blank cotton. The mass difference is recorded as 0.059 g in the dark and as 0.087 g under illumination. So, the solar evaporation rate difference is calculated as 0.42 kg/m^2^×h.

The solar evaporation efficiency is calculated using Equation S8, where ṁ is the solar evaporation rate difference, it is 1 kg/m^2^h.

| $Solar evaporation efficiency (\eta)=\frac{Total heat used to evaporate water}{Solar energy input}=\frac{ṁ\cdot\left( Q+h_{v} \right)}{P_{0}}$ | (Equation S8) |
| --- | --- |

Sensible heat, denoted by Q, is the heat absorbed by a substance resulting in a change in its temperature without a phase change. For this case, it is the temperature increase in the cotton surface, and it is calculated by the formula Q = C x ΔT where C is the specific heat of water (4185.5 J °C^-1^kg^-1^), ΔT is the difference between the initial (25.7 °C) and final temperature (29 °C) of the surface under the light, it is 3.3 °C. h_v_ is the enthalpy of vaporization since water is evaporated at 30 °C instead of 100 °C, vaporization enthalpy is calculated separately according to temperature ^[4]^ as 2430 J/g. P_0_ is 100 mW/cm^2^ as the power of the simulated sunlight. Thus, η is calculated as 68%.

**Table S1:** NIR effective bimorph photothermal soft actuator performances.

| **Material** | **Thickness (μm)** | **Light Intensity (W/cm^2^)** | **Time (s)** | **ΔT (°C)** | **Curvature (cm ^1^)** | **Reference** |
| --- | --- | --- | --- | --- | --- | --- |
| PDMS/BiSn | 170 | 3.7 | 2 | 85 | 0.63 | This work |
| PDMS/GNPs | 130 | 2.9 | 5 | 60 | 0.40 | ^[5]^ |
| PMMA/Au NRs-RGO | 25 | 0.5 | 0.5 | 35 | 2 | ^[6]^ |
| CNT-paper/BOPP | 76 | 0.2 | 10 | 38 | 1.6 | ^[7]^ |
| CNT/PE | 40 | 0.25 | 1.4 | 29 | 5 | ^[8]^ |
| GO/BOPP | 65 | 0.3 | 10 | 44 | 2.8 | ^[9]^ |
| TTA-COF/LLDPE | 11 | 0.2 | 3 | 11 | 2.35 | ^[10]^ |

*PDMS: Polydimethylsiloxane, BiSn: BiSn core-shell particles, GNPS: Graphene nanoplatelets, PMMA: Polymethylmethacrylate, Au NRS: Au nanorods, RGO: reduced graphene oxide, CNT: Carbon nanotubes, BOPP: Bioriented polypropylene, PE: Polyethylene.*


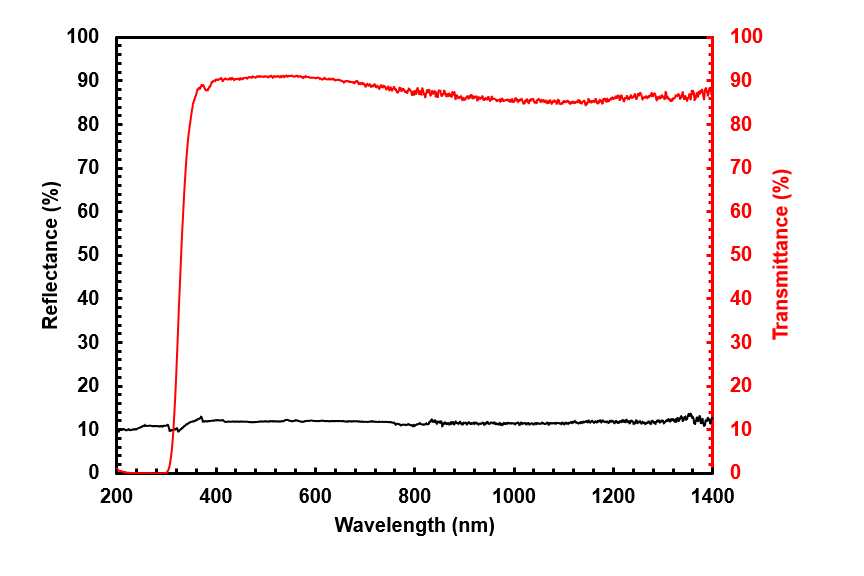


**Figure S8.** *Total transmittance (%) and total Reflectance (%) spectra of glass substrate.*

**Videos Showing NIR-sensitive PDMS/BiSn Photo Actuator Under Action**

**Video S1:** Curvature of the actuator (x2 speed, 60 fps, MP4)

**Video S2:** Dynamic shape adaptation of the actuator (x2 speed, 60 fps MP4)

**REFERENCES**

[1] Y. Cai, H. Zhu, Q. Shi, Y. Cheng, L. Chang, W. Huang, *iScience* **2022**, *25*, 103661.

[2] S. M. Karazi, I. U. Ahad, K. Y. Benyounis, in *Ref. Module Mater. Sci. Mater. Eng.*, Elsevier, **2017**, p. B9780128035818041497.

[3] “Solar Simulator,” can be found under https://www.ossila.com/products/solar-simulator, **n.d.**

[4] B. Henderson‐Sellers, *Q. J. R. Meteorol. Soc.* **1984**, *110*, 1186.

[5] W. Jiang, D. Niu, H. Liu, C. Wang, T. Zhao, L. Yin, Y. Shi, B. Chen, Y. Ding, B. Lu, *Adv. Funct. Mater.* **2014**, *24*, 7598.

[6] B. Han, Y. Zhang, L. Zhu, Y. Li, Z. Ma, Y. Liu, X. Zhang, X. Cao, Q. Chen, C. Qiu, H. Sun, *Adv. Mater.* **2019**, *31*, DOI 10.1002/adma.201806386.

[7] P. Zhou, L. Chen, L. Yao, M. Weng, W. Zhang, *Nanoscale* **2018**, *10*, 8422.

[8] L. Li, J. Meng, C. Hou, Q. Zhang, Y. Li, H. Yu, H. Wang, *ACS Appl. Mater. Interfaces* **2018**, *10*, 15122.

[9] L. Chen, M. Weng, P. Zhou, L. Zhang, Z. Huang, W. Zhang, *Nanoscale* **2017**, *9*, 9825.

[10] G. Chen, L. Chen, N. Li, J. Li, M. Huang, C. Gong, Y. Peng, *ACS Appl. Mater. Interfaces* **2023**, *15*, 36804.
